# Seroprevalence Study and Cross-Sectional Survey on COVID-19 for a Plan to Reopen the University of Alicante (Spain)

**DOI:** 10.3390/ijerph18041908

**Published:** 2021-02-16

**Authors:** Jose Tuells, Cecilia M. Egoavil, María Angeles Pena Pardo, Ana C. Montagud, Emilia Montagud, Pablo Caballero, Pedro Zapater, Joan Puig-Barberá, Jose Antonio Hurtado-Sanchez

**Affiliations:** 1 Department of Community Nursing, Preventive Medicine, Public Health and History of Science, University of Alicante, 03690 Alicante, Spain; 2 Clinical Pharmacology Unit, General University Hospital of Alicante, Institute for Health and Biomedical Research (ISABIAL Foundation), C/Pintor Baeza, 12, 03010 Alicante, Spain; pena_marpar@gva.es (M.A.P.P.); zapater_ped@gva.es (P.Z.); 3 Immunology Department, Fundación Jiménez Díaz University Hospital, 28040 Madrid, Spain; anacmontagud@gmail.com; 4 Primary Care Pharmacy Service, University Hospital of Torrevieja, 03186 Torrevieja, Alicante, Spain; emiliamontagud@gmail.com; 5 Vaccines Research Area FISABIO, 46020 Valencia, Spain; jpuigb55@gmail.com; 6 Faculty of Health Sciences, University of Alicante, 03690 Alicante, Spain; ja.hurtado@ua.es

**Keywords:** SARS-CoV-2, COVID-19, epidemiology, serological assay, cross-sectional study, higher education institutions, universities, undergraduates

## Abstract

The implementation of strategies to mitigate possible cases of COVID-19 were addressed at the University of Alicante for the safe reopening of the 2020/2021 academic year. To discover the prevalence of immunity against SARS-CoV-2, a study was designed using a rapid immunoassay test (carried out between 6 and 22 July 2020), and in addition a cross-sectional survey was conducted on risk factors, symptoms, predisposition for becoming vaccinated, and sources of information about COVID-19. A random sample, stratified by students, faculty, and administrative staff, was selected. The seroprevalence found was 2.64% (39/1479; 95% CI 1.8–3.4), and the adjusted seroprevalence was 2.89% (95% CI 2.1–3.7). The average age of the students was 23.2 years old, and 47.6 years old for staff. In relation to COVID-19, the following was found: 17.7% pauci-symptomatic, 1.3% symptomatic, 5.5% contact with cases, 4.9% confined, and 0.3% PCR positive. More than 90% complied with preventive measures. The proportion willing to receive the COVID-19 vaccine was 91%. Their sources of information were the Internet (74%) and television (70.1%). They requested that the university offer information (45.1%), training (27%), and provide Personal Protective Equipment (PPE) (26.3%). Lastly, 87.9% would repeat the test. A plan was established that included the follow-up of cases and contacts, random sample testing, training courses, bimodal teaching, a specific website, and the distribution of PPE.

## 1. Introduction

The devastating effect of COVID-19 has affected the public and private spheres, modifying our lifestyle and changing our relationship with our day-to-day environment. It has been already recognized that we are currently facing a new unprecedented pandemic with still unknown psycho-pathobiological aspects.

In Europe, Spain occupies a significant place among the countries that suffered the devastating effects of the disease, as it was one of the first countries affected after Italy [1,2]. This led to the declaration of a lockdown by the government on 14 March 2020, confining the population to their homes with strict measures to limit mobility. This situation, which lasted until 20 June of the same year, resulted in the closure of educational institutions, which did not resume their activity until after the summer, during the first weeks of September.

Due to the initial absence of an effective treatment or vaccine and of good diagnostic tools, this pandemic affected, and continues to affect, all areas of our society (including health, the economy, and education). Thus, proper patient management and limiting the spread of the virus is especially important [3]. Significant short, medium, and perhaps long-term consequences and disruptions from the pandemic appear to be inevitable, and these may become increasingly severe [4].

The World Bank estimated that, in April of 2020, universities and other tertiary educational institutions were closed in 175 countries and communities, and more than 220 million post-secondary education students had their studies ended or significantly disrupted due to COVID-19 [5]. Spain was one of the countries with the strictest conditions during the pandemic: leaving home was only allowed for essential needs [6], all universities were physically closed, and classes continued online with support from the Spanish government [7].

In recent months, the need to implement strategies to mitigate COVID-19 cases on university campuses for their safe reopening were addressed [8,9,10,11] in order to track possible outbreaks [12]. Faced with a possible skepticism or fear of returning to class due to risk perception [13], studies were carried out to simulate strategic models [14,15,16,17]. These studies showed that randomized testing, contact-tracing, and quarantining were important components of the strategy for containing campus outbreaks [13], as opposed to symptom-based screening [15,16], and also showed that these should be individualized according to each university [17].The feasibility of self-administered tests was verified [18], and the students’ knowledge and behaviors regarding COVID-19 [19] or the prevalence of symptoms were explored [20], especially in health sciences or medicine departments [21,22,23]. Reopening campuses and preventing outbreaks requires careful deliberation and the use of all scientific tools and advances available to develop plans and protocols that are appropriate to their jurisdiction [24].

In our universities, the main concern was what training and education would be like from now on in a pandemic context. Is it possible to continue with traditional classes? This question was even more important in the health-related professions, where the students must practice using physical contact. Therefore, controversy was guaranteed, as the seroprevalence of this population was not well known.

On the other hand, higher education institutions are uniquely placed to lead a coordinated scientific and educational movement to shape a future that supports both people and the planet [11,25] through education, research, and advocacy [26].

Seroprevalence has been extensively explored in patients tested by RT-PCR [27,28], but few studies have assessed seroprevalence in asymptomatic individuals. In Spain, Pollán et al. [29] carried out a large national and population-based sero-epidemiological study on the general population during the first wave of the pandemic (from April to May 2020), and found that most of the population appeared to have remained unexposed to SARS-CoV-2, even in areas with widespread virus circulation [30]. Other epidemiological data published were not disaggregated by age groups, sex, social group, autochthonous, imported, etc. Likewise, there was an insufficient number of detection tests carried out (PCR, ELISA, or rapid tests) [30,31].

At the time of our study, a rapid diagnostic test for COVID-19 with a strong scientific support was not available. Many of these were under development, and their features were being evaluated or had problems [32,33,34]. However, detecting antibodies against SARS-CoV-2 (IgG, IgM, and IgA) plays a complementary role in providing epidemiological information [35].

The Center of Disease Control and Prevention (CDC) recommended testing to diagnose COVID-19 as one key components of a comprehensive strategy, which should be used in conjunction with the promotion of behaviors that reduce spread, thereby maintaining healthy environments, maintaining healthy operations, and preparing for when someone becomes sick [24].

In this context, the University of Alicante (UA) decided to develop adequate plans and protocols to protect the university community and control the spread of SARS-CoV-2.

The aim of this study was to explore aspects related to the pandemic disease in our university community before the reopening of the 2020–2021 academic year. The aspects of special interest were associated with epidemiological surveillance for adopting strategies for a safe return to university.

## 2. Materials and Methods

### 2.1. Study Design

A seroprevalence cross-sectional design, with SARS-CoV-2 virus detection through a rapid immunoassay test, was utilized for the epidemiological study. The sample was selected from a representative random sample stratified by students, administrative staff, and faculty, and per academic program. The study was carried out, with a single test, organized over 13 days (6 July to 22 July 2020) before the reopening of the Alicante University after the summer vacation, with the maintenance of a strict protocol of protection for students and collaborators, and measures of social distancing. Simultaneously, a cross-sectional survey was carried out to determine how the university community had dealt with the pandemic crisis, which is explained below.

### 2.2. Sample Size Calculation

The UA sample frame was comprised of 28,304 members (25,635 students, 2286 professors, and 1383 administrative staff). The sample size was determined with equal probability of being selected, and to ensure sufficient precision for evaluating the percentage of participants immunized, assuming 5% of participants would be immunized, as reported by the National Sero-epidemiological Study of Spain [29]. We included 1500 individuals to allow this percentage to be estimated with a precision of at least 1.1%, assuming a 3% failure-rate.

### 2.3. Participants

Two ethics committees approved the study, the Ethics Committee for Research with Medicines of the Alicante Health Department-General Hospital, and the Ethics Committee of the University of Alicante.

After the approval of the study and maintenance of the confidentiality of the data, the Statistics Department from the University of Alicante (Data Processing Center) provided us with the list of randomized subjects, which included names, emails and/or telephone numbers. There were two additional randomized lists to replace participants’ absences. Randomized subjects were invited to participate in the study and visit the University of Alicante, Faculty of Health Sciences, to carry out the test. Participation was voluntary and without incentives.

### 2.4. Selection Criteria

The participants had to belong to the university community (students, faculty, or administrative staff) of the University of Alicante at the time of the study, whether or not they had become sick from the disease, and they had to have been selected for the study and given their written informed consent. Participants who declared immunodeficiency, immunosuppression treatment, or cancer were excluded.

### 2.5. Detection of SARS-CoV-2 Antibodies and Test Performance

The Cellex^®^ qSARS-CoV-2 IgG/IgM Rapid Test (Cellex Inc., Durham, NC, USA) was used [36,37]. This is a lateral flow immunoassay intended for the qualitative detection and differentiation of IgM and IgG antibodies against SARS-CoV-2 in serum, plasma, or whole blood specimens, producing results in 15–20 min. The manufacturer reports a sensitivity and specificity of 93.75% (95% CI: 88.06–97.26%), and 96.40% (95% CI: 92.26–97.78%), respectively, using RT-PCR as the gold standard. The test was verified by the Microbiology Service of the Valencia General Hospital Consortium, which has accreditation according to the UNE-EN ISO 15189: 2013 standard, which verified the characteristics of the test: a sensitivity of 87.80%; 99.9% specificity and an efficiency of 89.58% [38]. Twenty-two nursing students were trained to perform the test, proceeding according to the specifications recommended by the manufacturer and the Food and Drug Administration [36,37].

The whole blood sample was obtained directly from a finger-prick. Members of the research team supervised the reading of the final results. Positive cases required the assessment of at least two team members. All the controversial cases required a consensus of three members of the research team. For the antibody positivity report, we followed the manufacturer’s instructions [36].

### 2.6. Contact Tracing Assessment

A protocol was implemented in cases of a participant from the staff group (faculty and administrative) obtaining a positive test result in the study, including a thorough epidemiological investigation and contact tracing of colleagues who worked in the same facilities for 60 days before the test. Contact tracing started four days before the date indicated by the patient until the day the test was performed. For asymptomatic cases, the period of investigation was based on the date of the test. The contacts were invited to participate in the study. In the student group, positive cases were provided with information about confinement measures by a physician trained in COVID-19, and were then referred to their general practitioner.

### 2.7. Cross-Sectional Survey

A questionnaire designed ad hoc for the study was provided to the participants. It was previously evaluated by team members who gave their recommendations, ultimately approving the latest version by consensus. A pilot test was performed with 20 students from different programs other than Health Sciences to assess its comprehension. Participants in the pilot study were not taken into account for this analysis.

Google Forms was the online platform chosen for delivering the self-administered surveys. To maintain anonymity, the email addresses used were not collected. Participants accessed this with a bar-code provided by the person who performed the blood test.

The study’s purpose was explained, and the informed consent was signed before the finger blood sample was taken. While waiting for the result, the participants completed the survey in 10–15 min.

The questionnaire included items such as:

Socio-demographic variables (sex, age, nationality, group, and place of origin).

- Variables related to risk factors described for SARS-CoV-2 infection, such as chronic diseases, smoking, history of a previous infectious process in the last 12 months, use of drugs and previous vaccinations, as well as sun exposure and physical activity.
- Variables related to symptoms described for SARS-CoV-2 infection, contact with possible positive cases, and diagnosis of SARS-CoV-2 infection [39].
- To obtain an idea of the actions needed to be carried out in the following academic year at the University of Alicante, they were also asked about their usual form of travel to access the university, the reasons why they have left home during the confinement, and their predisposition towards receiving the influenza and/or SARS-CoV-2 vaccine in the autumn, as well as the individual protection measures taken.
- Lastly, they were asked about the sources of information that they regularly used to inform themselves about the pandemic, and possible expectations or demands for information and materials from the University.

### 2.8. Statistical Analysis

We estimated the seroprevalence as the proportion of participants who had a positive test result in the rapid test IgG band. Assuming that the test used was imperfect, the estimate was adjusted using the formula described by Greenland (1996) [40]. The result was weighted with the variables ‘sex’ and ‘staff/student’.

A descriptive statistical analysis was performed. The means and standard deviations were calculated. We used Student’s t-test to compare the means, and Chi-square tests and Fisher’s exact test (categorical variables) were performed to examine differences between groups in all the questionnaire items. To assess the association between the independent variables and the two populations, odds ratios (OR) and adjusted odds ratios (ORa) were performed using a logistic regression, and the 95% CI was calculated.

All the data were analyzed with the statistics program SPSS Statistics for Windows v20 (SPSS v20, IBM Corp., Armonk, NY, USA). The level of accepted statistical significance was *p* < 0.05.

### 2.9. Ethical Considerations

All the subjects received an informed consent form via email at the time of the invitation to participate in the study, to be read before the test. The study complied with the Ethical Principles for Human Research standards, and the study protocol (and the rest of the documents) were approved by two Ethics Committees.

The study was carried out following the Declaration of Helsinki and the EU Regulation 134 2016/679 on personal data handling. Participation was completely voluntary and all the participants were asked to provide their informed consent in writing and to sign this before the blood sample was taken and before obtaining the barcode. The participants were informed that all the information collected would be anonymous and treated as confidential. The participants could not be identified from the collected material.

## 3. Results

### 3.1. Prevalence Study

1479 subjects were studied from the randomized sample. We could not reach out to 21 students from the seroprevalence study, and 7 of them refused to participate, because they lived far from the UA. Therefore, the response ratio was 0.99.

Overall, the seroprevalence found for the university community of Alicante in the study period from 6 July to 22 July 2020, was 39/1479 (2.64%; 95% CI 1.8–3.4) through the use of the lateral flow immuno-assay test. The adjusted seroprevalence, according to the data from the Valencian study [38], was 2.89% (95% CI 2.1–3.7) (Table 1).

### 3.2. Population Characteristics and Survey Response

The cross-sectional study sample comprised of 1359 subjects, and the response rate for the survey was 0.92 (1359/1479). Among the participants who answered the questionnaire, 1021 (75.1%) were students, and 338 (24.9%) were faculty and staff members. Of these, 919 (67.6%) were women (male: female ratio = 0.47), and the mean age was 23.2 years old (±6.4) in the student group, and 47.6 (±9.5) in the staff group. Only 1.6% (22/1359) were foreigners (Table 2).

Blood group A was predominant at 48.2% (509/1056), and 19.1% (260/1359) confirmed suffering from a chronic disease. Globally, the most reported chronic diseases were allergy/asthma (4.8%; 65/1359), cardiovascular (4.1%; 56/1359), and metabolic diseases (3.9%; 53/1359). Allergies were more frequent in the group of students (5%; 51/1021), while in the staff group, cardiovascular diseases were more frequent (10.4%; 35/338). None of the participants declared immunodeficiency, immunosuppression treatment, or cancer.

There were no differences in smoking between both groups. Still, we found significant differences in the mean number of cigarettes/day between the populations: 6 (±4.77) in the student group, and 10.2 (±7.28) in the staff sample (*p* = 0.001).

Table 3 shows the distribution of anti-SARS CoV-2 results in students and faculty and administrative staff. The high prevalence in female students should be noted, as only 16% (4/25) of the positive tests results were from male students. This could be associated with the male/female ratio of the students. There were four participants who reported a previous positive PCR SARS-CoV-2 test; three of them had positive antibody tests. Only 28% (7/25) of the positive cases reported some prior illness with respiratory symptoms during the 14 days before the study.

Table 4 shows the characteristics of the population related to the symptoms described for SARS-CoV-2 infection. Only 18.9% (258/1359) reported symptoms in the two–three weeks before the date of the serological study, and 14 of them reported ageusia and/or anosmia (5.4%; 14/258). 

During the confinement period (between March and April), most participants cohabited with another three to five people (55.2%; 750/1301). In addition, 1.5% (20/1359) affirmed to living at home with a positive case of SARS-CoV-2, and 4.9% (66/1359) remained strictly confined at home due to medical recommendations.

Additionally, 3.1% (42/1359) had taken a PCR test for SARS-CoV-2, and four were positive. Similarly, 2.5% (35/1359) had taken a serological test, and only one resulted as positive.

In regard to suffering infections in the 12 months prior to the test, 12.6% (171/1359) had had some type of infection. The most frequent, 7.7% (105/1359), were related to respiratory infections, and there was a statistically significant difference between the groups of students and staff.

Lastly, 15.8% (215/1359) had received a vaccine in the 12 months before the survey. The most frequent vaccine was against influenza (9.1%; 123/1359), followed by the meningococcal (2.1%; 29/1359) vaccine. Allergy vaccines had been received by 1.5%.

Some variables related to attitudes towards SARS-CoV-2 infection were explored, which are shown in Table 5. Most of the participants declared using hydrogel (97.6%; 1324/1357) and hand washing (96.6%; 1309/1355) as the main protection measure; a mask was used by 94% (1277/1358), and physical distancing by 88.5% (1198/1353).

Only 2.7% (37/1359) affirmed not having any reasons for leaving their home during the confinement, mainly students. The majority (61%; 829/1359) recognized at least two reasons for leaving home, such as to shop (72%; 978/1359), or go for a walk (63.6% 864/1359), with no differences between the two groups.

We found that 70.5% (958/1359) of the subjects did some type of physical exercise, with an average of 5.8 ± 7.8 h/week, without statistically significant differences between both groups. The primary activity was walking 18.7% (214/1145), and 33% (429/1272) performed indoor activities (such as gymnastics, cardiovascular, or bodybuilding exercises). An average of 2.5 ± 2.1 h of sun exposure was reported, with a statistically significant difference between both groups (2.7 ± 1.9 students and 1.9 ± 2.6 staff *p* = 0.005).

We also found that if a COVID-19 vaccine were available, 91% (1210/1329) would be willing to use it, while only 31% (407/1312) would obtain a flu vaccine in the fall.

As for how the participants traveled to the University campus during the academic period, we found that 91% (1235/1359) generally arrived with a motorized vehicle, and only 9.1% (124/1359) attested arriving on foot or by bicycle (Table 6). A private/individual car (66.6%; 905/1359) was the most common vehicle used by both groups, followed by the bus (30.5%; 415/1359).

Both groups used the internet and television as the media of choice for obtaining information about the pandemic and the SARS-Cov-2 infection; the primary demand on the University was accessible information on the university’s website and training courses. Lastly, the majority would repeat (87.9%; 1195/1359) the rapid test in a new study.

### 3.3. Contact Tracing Assessment

We listed 103 persons from the university environment in the contact-tracing study of 14 positive cases among the randomized staff members. We reached out to 94 individuals and detected two secondary cases with an infection risk of 2.1% (95% CI, 0.5%–1.2%).

## 4. Discussion

In our study, the prevalence of IgG antibodies against SARS-CoV-2 from the 6th to the 22nd of July 2020, in a randomized sample of students and faculty and administrative staff from the University of Alicante, was 2.64%. These data are in agreement with the 2.4% observed in our region in the first wave of the ENE-COVID study [29], a population-based study. Our findings assessed the situation during the first wave, as the pandemic started to improve in early July. Afterwards, the second wave would begin, which has not yet remitted.

Tilley et al. [41] in the USA (Los Angeles), and Tsitsilonis et al. [42] in Greece (Athens), obtained similar results. The seroprevalences of the university community and in the general population were very similar.

Other seroprevalence studies have been conducted, and the efficacy of different testing strategies in higher education institutions is still being evaluated. In their study, Blaisdell et al. [43] suggested that a two-phased universal testing strategy may be effective in minimizing transmission, and the experiences of the University of Texas and North Carolina University highlights the potential for rapid transmission on campus [12,44,45]. Gillam et al. [18] proposed that repeated self-testing for COVID-19 using PCR is feasible and acceptable for a university population.

To our knowledge, this study is the first in Spain with an academic population. We have to add that there was a good acceptance rate to participate among those invited.

We found no differences in seroprevalence between students and faculty and staff, although not everyone who is infected with COVID-19 will develop an immune response [44].

Other studies have shown that universal testing may have a significant impact on the control of the virus, depending on the ability of the location to implement other control measures [44], but universal testing and testing symptomatic people are not well studied as testing programs for COVID-19 at a university campus [18], and the type of test that would be the most useful in the university context considered is still unknown.

There is still no consensus on the forms and types of approach that could be used to evaluate students for COVID-19 when returning to the university campus [16]. In general, different institutions have different proposals regarding the initial tests [3,9,19,24,33,46,47]. In Spain there is no consensus among the different regions, and at the central government level only very general recommendations have been made. Even now, scientific publications are only just starting to appear with discussions, considerations, and modeling of possible preventive strategies and their cost-effectiveness when it comes to reopening the university [8,14,15,48].

In our study, we found statistically significant differences between students and the university staff in almost all the factors explored, but it is remarkable that smoking was similar in both groups, so that both have the same risks associated with smoking. It was confirmed that the asymptomatic and pauci-symptomatic states, even if self-reported, were very frequent (they represented nearly 80% of the cases). Therefore, it is important to identify this quickly to prevent infection. While most of the students spent their lockdown in the family home with three to five family members, only 1.5% of them claimed to live with a SARS-CoV-2 positive case. These results are possible given the prevalence shown by the Ministry of Health at that time [29].

From the sample, 3% reported having undergone a PCR test for SARS-CoV-2, and of these four were positive. Additionally, 2.5% had done a serological test, and only one had received a positive result. The problems with the SARS-CoV-2 diagnostic tests (both PCR and antibodies test) are mainly the direct and indirect costs, and the variability in their sensitivity and specificity. This has been demonstrated and assessed in different studies, and reviewed in a systematic metanalysis [33].

Among all the positive cases, only 23% (9/39) had symptoms. This finding is important in a university campus; if large numbers of the population are asymptomatic or have mildly symptomatic infections, seroprevalence estimation studies may underrepresent the prior incidence of the disease.

In addition, important aspects of this infection are not yet known, such as the duration of immunity or the number of antibodies that are necessary to be protected, and whether or not reinfection and cross-reactivity with human endemic coronavirus are possible [27].

Approximately 95% of the participants declared following proper prevention methods against the spread of the virus, such as washing their hands regularly, wearing a face mask, following social distancing measures, and using hydroalcoholic gel. This result was higher than other studies conducted at about the time of the present study [19,21,23].

Most students and staff (91%) stated that they would receive a COVID-19 vaccine immediately if available; Chesser [19] found lower data in a university population (68%), but this contrasts with the 31% of all participants who would get the flu vaccine, and this could be the effect of a greater perception of severity and uncertainty produced by COVID-19. 

The most accessed sources for current COVID-19 information were the internet (79%) and television (70.3%), among students, in agreement with previous studies [19,23]. The significant differences found regarding the use of information sources between students and staff groups were remarkable; the students preferred social media, and the faculty and administrative staff preferred the digital press as they type of media used to inform themselves about the pandemic topics. This information could help health educators to develop communication strategies to improve their reach and the knowledge of the population [19]. In addition, students’ demands showed the need for more information on the University’s website, more training courses, and individual training teams. This is an indication of the interest in our community in being up-to-date on the subject of COVID 19, as 87.9% indicated that they would be willing to repeat the test, which indicates the majority acceptance of this initiative and their favorable predisposition towards it.

Considering that clear leadership was necessary to confront this emergency [11], a multidisciplinary working group was created and coordinated by a medical epidemiologist appointed by the University Rector on 1 May 2020. Each of the faculties provided a person who would be responsible for COVID-19 related matters, who joined the working group. A single institutional email on COVID-19 and a contact telephone number were established [11].

Following the guidelines recommended by the health and academic authorities, the University of Alicante (UA) prepared, on 10 July 2020, a Risk Prevention Guidelines against COVID-19, with all the necessary prevention measures to be implemented in September of the 2020/2021 academic year, with guarantees of a safe reopening according to the epidemiological situation.

Individual preventive measures (mandatory use of face masks, physical distance, hand hygiene), and collective measures (natural ventilation, disinfection of classrooms, signage, isolation rooms) were encouraged; short training courses on COVID-19 epidemiology and prevention were held, aimed at different groups in the community; people with vulnerabilities were specified to telework, and a bimodal teaching method was chosen.

**Figure 1:**
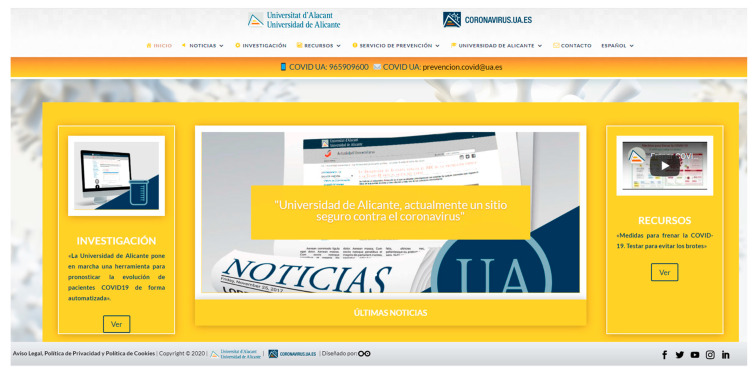
Coronavirus website of the University of Alicante: www.coronavirus.ua.es.

A Webpage on Coronavirus (Figure 1) was also created with its own and external content on training and dissemination. A COVID-19 Unit formed by health workers was created for the epidemiological surveillance and monitoring of cases and contacts. This unit, from 3 September to 18 December, tracked a total of 731 individuals with 200 positive PCR cases (152 students, 30 professors, 18 staff), and 531 close or suspicious contacts (276, 83, 172). In both cases, the detected persons were advised to stay in isolation (positive) or quarantine (close contact) for 10 days. They were followed-up by telephone to check their health status and to verify the absence of symptoms and positive tests. Random screening was and is being carried out by testing a random sample of the university population [8].

The antibody test was repeated in 37 of the 39 cases with a positive IgG result in July, which confirmed the same results, so these individuals had maintained their immunity throughout these past six months.

We could point out some limitations of our study. It was carried out in a specific population, thus the extrapolation of the results cannot be applied directly to the general population. However, the study serves to obtain preliminary information for future studies that are specifically designed, and we consider it representative of the university community. Hence, the findings were useful for decision-making during the reopening of the campus phase. Another limitation is inherent to the test. The test used to detect antibodies against SARS-CoV-2 in vitro is a qualitative test that does not determine the quantitative value or the rate of the increased levels of antibodies to SARS-CoV-2. This limits the scope of the estimation of the immunological status of the population, as a positive result with an insufficient amount of antibodies to protect against re-infection could be the case.

## 5. Conclusions

Different strategies based on testing must be implemented as part of a broader COVID-19 prevention plan that must be developed by higher education institutions, which can be adapted according to increases in knowledge, and incorporating new preventive measures such as vaccination.

Vaccines against COVID-19 are a reality; in December, vaccination was set to begin in several countries. The 27 member states of the European Union begun vaccinating on 27 December 2020, a symbolic date to reaffirm unity of action, and coinciding with the declaration of the first cases of the disease a year prior. However, this encouraging news does not allow us to lower our guard, as the number of cases and deaths from COVID-19 continues to rise.

Likewise, the higher education institutions’ population is not part of the most vulnerable groups and is not among those who will have priority access to vaccination. Therefore, it will be necessary to continue to maintain active epidemiological surveillance and follow-up of cases and contacts, carry out screening strategies, decide a flexible teaching model for the coming months, persist in training in individual and collective preventive measures, and take the opportunity to explain and describe the advantages of vaccination to the university community, eliminating any doubts about its efficacy and safety.

## Figures and Tables

**Table 1:** Seroprevalence of immunity against SARS-CoV-2 in the University community.

Prevalence Study	Prevalence	95% CI	
		LL (%)	UL (%)
Weighted Prevalence	2.64	1.84	3.43
Adjusted prevalence (se: 87.80%; sp: 99.9%) *	2.89	2.06	3.73

* Valencian study [38]; se = sensitivity; sp = specificity; LL = Lower Limit; UP = Upper Limit.

**Table 2:** Demographics and clinical characteristics of the sample.

Variable	Students *N* (%)	Staff *N* (%)	Total *N* (%)	OR (95% CI)	*p*
Overall Test Result					
Positive	25 (2.4)	14 (4.1)	39 (2.9)	1.7 (0.9–3.4)	NS
Negative	996 (97.6)	324 (95.9)	1320 (97.1)		
Sex					
Female	733 (71.8)	186 (55.0)	919 (67.6)	0.5 (0.4–0.6)	<0.001
Male	288 (28.2)	152 (45)	440 (32.4)		
Nationality					
Spanish	1002 (98.1)	335 (99.1)	1337 (98.4)	0.5 (0.1–1.6)	NS
Other	19(1.9)	3(0.9)	22(1.6)		
Blood group (*n* = 1056) **					
A	390 (48.7)	119 (46.7)	509 (48.2)	Ref	
AB	28 (3.5)	10 (3.9)	38 (3.6)	0.9 (0.7–1.2)	0.524
B	68 (8.5)	20 (7.8)	88 (8.3)	1.1 (0.5–2.3)	0.877
O	315 (39.3)	106 (41.6)	421 (39.9)	0.9 (05–1.5)	0.628
Smoking *					
Yes	118 (11.6)	46 (13.6)	164 (12.1)	1.2 (0.8–1.7)	NS
No	903 (88.4)	292 (86.4)	1195 (87.9)		
Chronic diseases					
Yes	153 (15.0)	107 (31.7)	260 (19.1)	2.6 (2–3.5)	<0.001
No	868 (85.0)	231 (68.3)	1099 (80.9)		

* Smoking (number of cigarettes/day). Age (years). NS (Not Significant). ** Blood group declared by participants. CI = confidence interval. Ref = reference.

**Table 3:** Prevalence of anti-SARS CoV-2 in students and faculty and administrative staff.

Variable, *N* (%)	Antibody Negative	Antibody Positive	Total	OR (95% CI)	*p*
**Students (*N* = 1021)**	**996**	**25**	**1021**		
Age (years), mean (SD)	23.2 (6.4)	22.9 (8.3)			NS
Gender, Female	712 (71.5)	21 (84)	733 (71.8)	2.1 (0.7–6.2)	NS
History PCR SARS-CoV-2 (+)	1 (1.0)	2 (8)	3 (2.9)	0.02 (0.3)	0.02
Smoking	116 (11.6)	2 (8)	118 (11.6)	0.7 (0.2–2.8)	NS
Chronic diseases	150 (15.1)	3 (12)	153 (15)	0.8 (0.2–2.6)	
Self-reported symptoms ▪	224 (22.5)	7 (28.0)	219 (21.5)	1.3 (0.6–3.2)	NS
Reported contact with 1 confirmed (PCR) COVID-19	14 (1.4)	3 (12.0)	17 (1.7)	9.6 (2.6–35.6)	0.007
Infection disease 12 month previous	136 (13.7)	5 (20.0)	141 (13.8)	1.6 (0.5–4.2)	NS
Vaccination 12 months previous	158 (15.9)	6 (24.0)	164 (16.1)	1.7 (0.6–4.2)	NS
**Staff (*N* = 338)**	**324**	**14**	**338**		
Age (years), mean (SD)	47.5 (9.4)	51.9 (11.8)			NS
Gender, Female	180 (55.6)	6 (42.7)	186 (55.0)	0.6 (0.2–1.8)	NS
History PCR SARS-CoV-2 (+)	0 (0)	1 (7.1)	7 (2.0)		0.001
Smoking	44 (13.6)	2 (14.3)	46 (13.6)	1.1 (0.2–4.9)	NS
Chronic diseases	99 (69.2)	8 (80.0)	107 (69.9)	3 (1.02–9)	0.036
Self-reported symptoms	48 (14.8)	2 (14.3)	39 (11.5)	0.9 (0.2–4.5)	0.037
Reported contact with 1 confirmed (PCR) COVID-19	2 (0.6)	1 (7.1)	3 (0.9)	12.3 (1.1–14)	NS
Infection disease 12 month previous	27 (8.3)	3 (21.4)	30 (8.9)	3 (0.7–11.4)	NS
Vaccination 12 months previous	51 (15.8)	1 (7.1)	52 (15.4)	0.4 (.05–3.2)	NS

▪ pauci-symptomatic (1–2 symptoms without anosmia or ageusia), and symptomatic (anosmia or ageusia, or at least three symptoms among fever; chills; severe tiredness; sore throat; cough; shortness of breath; headache; or nausea, vomiting, or diarrhea) during the 14 days before study.

**Table 4:** Characteristics related to SARS-CoV-2 infection symptoms (*n* = 1359).

Variable, N (%)	Students *N* (%)	Staff *N* (%)	Total *N* (%)	OR (95% CI)	*p*
Self-reported symptoms †					
Asymptomatic	802 (78.6)	299 (88.5)	1101 (81)	Ref	
Pauci-symptomatic	204 (20)	36 (10.7)	240 (17.7)	0.5 (0.3–0.7)	<0.001
Symptomatic ≤14 days before study	15 (1.5)	3 (0.9)	18 (1.3)	0.5 (0.1–1.9)	0.328
Household size, residents (*n* = 1301)					
One	133 (13.4)	92 (30.2)	225 (17.3)	Ref	
Two	214 (21.5)	83 (27.2)	297 (22.8)	0.7 (0.4–0.8)	0.002
Three to five	622 (62.4)	128 (42)	750 (57.6)	0.3 (0.2–0.4)	<0.001
Six or more	27 (2.7)	2 (0.7)	29 (2.2)	0.1 (0.03–0.5)	0.003
Household member with confirmed case					
No contact	1004 (98.3)	335 (99.1)	1339 (98.5)	0.53 (0.15–1.82)	NS
Household member	17 (1.7)	3 (0.9)	20 (1.5)		
Household member with symptomatic person					
No contact	828 (81.1)	278 (82.2)	1106 (81.4)	0.93 (0.67–1.28)	NS
Household member	193 (18.9)	60 (17.8)	253 (18.6)		
Contact with confirmed case last month					
Contact	59 (5.8)	15 (4.5)	74 (5.5)	0.76 (0.43–1.36)	NS
No contact	958 (94.2)	320 (95.5)	1278 (94.5)		
Confined during the last month					
Confined	55 (5.4)	11 (3.3)	66 (4.9)	0.59 (0.31–1.14)	NS
No	966 (94.6)	327 (96.7)	1293 (95.1)		
Self-reported PCR status					
Never done	991 (97.1)	326 (96.4)	1317 (96.9)	Ref	
Negative	27 (2.6)	11 (3.3)	38 (2.8)	0.8 (0.62–2.4)	NS
Positive (>14 days before study visit)	3 (0.3)	1 (0.3)	4 (0.3)	0.8 (0.3–1.6)	
Self-reported serologic status					
Never done	992 (96.8)	332 (97.9)	1324 (97.1)	Ref	
Negative	32 (3.1)	7 (2.1)	34 (2.9)	0	NS
Positive (>14 days before study visit)	1 (0.1)	0(0)	1 (0.1)	1.5 (0.6–3.5)	
Infection disease 12 month prior					
No	880 (86.2)	308 (91.1)	1188 (87.4)	Ref	
Respiratory diseases	81 (7.9)	24 (7.1)	105 (7.7)	0.8 (0.5–1.4)	0.491
No respiratory diseases	60 (5.9)	6 (1.8)	66 (4.9)	0.3 (0.1–0.7)	0.004
Vaccination 12 months prior					
No	857 (83.9)	287 (84.9)	1144 (84.2)	0.93 (0.66–1.31)	NS
Yes	164 (16.1)	51 (15.1)	215 (15.8)		

† Asymptomatic (no symptoms), pauci-symptomatic (1–2 symptoms without anosmia or ageusia), and symptomatic (anosmia or ageusia, or at least three symptoms among fever; chills; severe tiredness; sore throat; cough; shortness of breath; headache; or nausea, vomiting, or diarrhea). Ref = reference.

**Table 5:** Attitudes towards SARS-CoV-2 infection.

Variable	Students *N* (%)	Staff *N* (%)	Total *N* (%)	OR (95% CI)	*p*
Individual protection measures					
Wearing a face mask (*n* = 1358)					
No	64 (6.3)	17 (5)	81 (6)	1.3 (0.7–2.2)	NS
Yes	956 (93.7)	321 (95)	1277 (94)		
Wash hands regularly (*n* = 1355)					
No	43 (4.2)	3 (0.9)	46 (3.4)	4.9 (1.5–15.9)	0.003
Yes	975 (95.8)	334 (99.1)	1309 (96.6)		
Use hydroalcoholic gel (*n* = 1357)					
No	23 (2.3)	10 (3)	33 (2.4)	0.8 (0.4–1.6)	NS
Yes	997 (97.7)	327 (97)	1324 (97.6)		
Follow social distancing (*n* = 1353)					
No	150 (14.7)	5 (1.5)	155 (11.5)	11.5 (4.7–28.2)	
Yes	867 (85.3)	331 (98.5)	1198 (88.5)		
Reasons for departure * (*n* = 1359)					
Remain confined	31 (3)	6 (1.8)	37 (2.7)	Ref	
2 reasons for leaving	622 (60.9)	207 (61.2)	829 (61)	1.7 (0.7–4.2)	0.232
>3 reasons for leaving	368 (36)	125 (37)	493 (36.3)	1.8 (0.7–4.3)	0.219
Perform physical exercise (*n* = 1357)					
No	326 (31.9)	75 (22.2)	401 (29.5)	1.6 (1.2–2.2)	0.001
Yes	695 (68.1)	263 (77.8)	958 (70.5)		
Would get COVID-19 vaccine (*n* = 1329)					
No	90 (8.9)	29 (9)	119 (9)	1 (0.6–1.5)	NS
Yes	916 (91.1)	294 (91)	1210 (91)		
Is going to get flu vaccines (*n* = 1312)					
No	701 (71.4)	204 (61.8)	905 (69)	0.6 (0.5–0.8)	0.001
Yes	281 (28.6)	126 (38.2)	407 (31)		

Ref = reference.

**Table 6:** Mobility, sources of information and expectations.

Variable	Students *N* (%)	Staff *N* (%)	Total *N* (%)	OR (95% CI)	*p*
Ways to access to the university					
On foot/by bicycle	101 (9.9)	23 (6.8)	124 (9.1)	1.5 (0.9–2.4)	NS
Motorized vehicle	920 (90.1)	315 (93.2)	1235 (90.9)		
Use of means of transport					
Private car	623 (61)	282 (83.4)	905 (66.6)	3.2 (2.4–4.4)	<0.001
Bus	388 (38)	27 (8)	415 (30.5)	0.1 (0.1–0.2)	<0.001
On foot	171 (16.7)	30 (8.9)	201 (14.8)	0.5 (0.3–0.7)	<0.001
Shared vehicle	168 (16.5)	9 (2.7)	177 (13)	0.1 (0.1–0.3)	<0.001
Motorcycle	20 (2)	17 (5)	37 (2.7)	2.7 (1.4–5.1)	0.003
Bicycle	37 (3.6)	20 (5.9)	57 (4.2)	1.7 (1–2.9)	NS
Information media					
Internet	807 (79)	199 (58.9)	1006 (74)	0.4 (0.3–0.5)	<0.001
Television	718 (70.3)	235 (69.5)	953 (70.1)	1 (0.7–1.3)	NS
Social networks	561 (54.9)	75 (22.2)	636 (46.8)	0.2 (0.2–0.3)	<0.001
Digital press	413 (40.5)	182 (53.8)	595 (43.8)	1.7 (1.3–2.2)	<0.001
Official website	489 (47.9)	105 (31.1)	594 (43.7)	0.5 (0.4–0.6)	<0.001
Radio	153 (15)	98 (29)	251 (18.5)	2.3 (1.7–3.1)	<0.001
Non-official website	55 (5.4)	8 (2.4)	63 (4.6)	0.4 (0.2–0.9)	0.02
Expectations towards the University regarding COVID-19					
None	17 (1.7)	5 (1.5)	22 (1.6)	Ref	
Provide individual training teams	180 (17.6)	177 (52.4)	357 (26.3)	3.3 (1.2–9.3)	0.020
Information accessible from a website	500 (49)	113 (33.4)	613 (45.1)	0.8 (0.3–2.1)	0.612
Specific training on the pandemic	324 (31.7)	43 (12.7)	367 (27)	0.5 (0.2–1.3)	0.130
Would repeat the test					
No	8 (0.8)	0 (0)	8 (0.6)		
Yes	880 (86.2)	315 (93.2)	1195 (87.9)	Ref	
Maybe	133 (13)	23 (6.8)	156 (11.5)	0.5 (0.3–0.8)	0.002

Ref = reference.

## Data Availability

The data presented in this study are available on request from the corresponding author. The data are not publicly available due to privacy reasons.
